# Differential effects of community health worker visits across social and economic groups in Uttar Pradesh, India: a link between social inequities and health disparities

**DOI:** 10.1186/s12939-017-0538-6

**Published:** 2017-03-07

**Authors:** Aparna Seth, Shweta Tomar, Kultar Singh, Dharmendra Chandurkar, Amit Chakraverty, Arnab Dey, Arup K. Das, Katherine Hay, Niranjan Saggurti, Sabrina Boyce, Anita Raj, Jay G. Silverman

**Affiliations:** 1 Woodrow Wilson School of Public and International Affairs, Princeton University, Princeton, NJ USA; 2 Sambodhi Research and Communications Pvt. Ltd., Noida, Uttar Pradesh India; 3 Bill and Melinda Gates Foundation, New Delhi, India; 4 Population Council, New Delhi, India; 5 Center on Gender Equity and Health, Division of Global Public Health, University of California, San Diego School of Medicine, San Diego, CA USA; 6 India Health Action Trust, Lucknow, India

**Keywords:** Maternal health, Neonatal mortality, Health inequities, Community health workers, Home visits

## Abstract

**Background:**

Uttar Pradesh (UP) accounts for the largest number of neonatal deaths in India. This study explores potential socio-economic inequities in household-level contacts by community health workers (CHWs) and whether the effects of such household-level contacts on receipt of health services differ across populations in this state.

**Methods:**

A multistage sampling design identified live births in the last 12 months across the 25 highest-risk districts of UP (*N* = 4912). Regression models described the relations between household demographics (caste, religion, wealth, literacy) and CHW contact, and interactions of demographics and CHW contact in predicting health service utilization (> = 4 antenatal care (ANC) visits, facility delivery, modern contraceptive use).

**Results:**

No differences were found in likelihood of CHW contact based on caste, religion, wealth or literacy. Associations of CHW contact with receipt of ANC and facility delivery were significantly affected by religion, wealth and literacy. CHW contact increased the odds of 4 or more ANC visits only among non-Muslim women, increased the odds of both four or more ANC visits and facility delivery only among lower wealth women, increased the odds of facility delivery to a greater degree among illiterate vs. literate women.

**Conclusion:**

CHW visits play a vital role in promoting utilization of critical maternal health services in UP. However, significant social inequities exist in associations of CHW visits with such service utilization. Research to clarify these inequities, as well as training for CHWs to address potential biases in the qualities or quantity of their visits based on household socio-economic characteristics is recommended.

## Background

Despite the progress in provision of health care access, India’s health system continues to struggle with the challenge of addressing inequalities in utilization of maternal and reproductive health services based on demographic characteristics [1–3]. Key questions include whether continuing inequities stem from the potential interplay of social determinants and the likelihood and utility of household-level contacts by health care workers, as these interactions are the basis for engagement with the health system. To answer these questions, potential biases of health care workers related to the demographic profiles of households visited and potential differential impact of those visits on increasing the likelihood of such beneficiaries seeking or receiving care must be examined. Such data are critical to addressing and improving health outcomes among the most marginalized groups and, thus, to reducing disparities in maternal and child mortality in India.

India, with a neonatal mortality rate (NMR) of 29 deaths per 1000 live births [4], accounts for more than a quarter of these deaths globally [5]. Uttar Pradesh (UP) is both the most populous state in India (204.2 M people) and suffers one of the highest NMR (50) in the country [6]. In the Indian and other national contexts, studies suggest the protective effects of antenatal care, institutional delivery and use of modern contraceptives (via smaller numbers and more widely spaced births) on infant and maternal mortality [7–12]. Neonatal and maternal survival are also positively correlated with increasing density of community health workers [13, 14]; in rural India, such workers are called ASHAs (accredited social health activists). ASHAs provide a variety of services to households, including the delivery of basic health care, health education, and promoting uptake of facility-based health care, particularly antenatal care and facility delivery [15].

Research has demonstrated that ASHA programs can be effective in reducing maternal and child mortality in low-income settings in India by providing such critical interventions [16–20]. However, there exist disparities in utilization of maternal and reproductive health services (e.g., antenatal care, facility delivery and modern spacing contraception) based on socio-demographic characteristic of beneficiaries. Disparities in maternal care utilization have been observed related to caste, religion, education and wealth [21–23]. These reduced levels of care are, at least in part, responsible for the relatively higher vulnerability of neonates from these populations regarding morbidity and mortality [24]. These care-related disparities are meant to be addressed in India via ASHAs reaching all households of pregnant women, particularly the most marginalized, and facilitating their utilization of critical health services. However, little is known about potential inequities in provision of services by ASHAs based on household demographics, or whether the effects of such household-level contacts differ across these populations. Both questions are critical to understanding the factors responsible or ongoing inequities in reproductive, maternal and child health and survival. This study attempts to fill these gaps in knowledge via analyses of ASHA contact and health service utilization data from a large population-based sample of recent births from Uttar Pradesh. The primary objectives of this study were to assess differences in the likelihood of ASHA contact and differences in the relationship of such contacts to receipt of health services based on household demographics regarding social and economic marginalization (i.e., caste, religion, literacy and wealth). Findings will inform prioritization and modification of current health care policies and other structures related to improving the reach and qualities of household-level contact by community health workers to reduce inequities in health care utilization and, thus, promote maternal and child survival among the most marginalized in Indian society.

## Methods

Data analyzed in the current study were collected as part of the baseline for the evaluation of the Uttar Pradesh Technical Support Unit (UPTSU) intervention. All the districts of the state were ranked on a composite index of indicators which comprised of maternal mortality ratio (MMR), percentage of safe deliveries, infant mortality rate (IMR), percentage of children 12–23 months fully immunized, total fertility rate (TFR) and contraceptive prevalence rate (CPR) – modern method, with the 25 most poorly performing districts designated as High Priority Districts (HPDs) [25]. Effect sizes seen in previous studies involving the outcome of receipt of three or more antenatal care visits were used to calculate the required sample size for the current study. The baseline estimate for this indicator in rural UP was taken from the Annual Health Survey (AHS) 2011–12 [6].

A multistage sampling design was used to create a representative sample of live births in the last 12 months from the 25 HPDs of Uttar Pradesh. At the first stage, four blocks (areas representing approximately 100,000 residents; range of approximately 10–30 blocks per district) were selected within each district representing those with highest (two blocks) and lowest (two blocks) numbers of facility-based deliveries. These 100 blocks in the HPDs were designated as treatment areas, i.e., those receiving a series of interventions to maximize the reach and impact of government health services, in these poorest performing districts. An additional 50 blocks were randomly selected from 100 blocks within these same districts (four per district) identified based on matching demographics and health indicators to allow assessment of effects on blocks not receiving the interventions but within intervention districts. At the second stage, within each of the 150 selected blocks, three ASHA catchment areas (representing approximately 1000 households; 150–450 ASHA areas per block) were randomly selected, resulting in a sample of a total of 450 ASHA areas at this second stage. At the third stage, a census of all the households from each selected ASHA area was conducted to identify all women who had a live birth in last 12 months. Of the 8474 children born alive in the preceding 12 months, 5291 were randomly selected to participate in the study; 4912 consented to be interviewed (92.2% participation rate). Data were weighted based on the sampling design to produce estimates representative of the selected blocks within the 25 highest-need districts of UP. Data included in the current study were collected prior to implementation of health systems interventions, and intervention-related assignment is not considered in the current analyses.

Survey interviews with participating women were conducted between June and October of 2014 by female research staff. Study protocols were reviewed and approved by the National Rural Health Mission (NRHM) of Uttar Pradesh, PHS-ERB (an independent ethical review board) and the *Health Ministry Screening Committee’s (HMSC) Indian Council for Medical Research (ICMR).* These protocols were also registered with the Clinical Trial Registry – India (CTRI/2015/09/006219).

### Measures

The four indicators of socio-economic inequity included caste, religion, household wealth and women’s literacy status. Households were categorized into three caste categories; from most to least marginalized, they are Scheduled Caste/Scheduled Tribe (SC/ST), Other Backward Caste (OBC) and General Caste (GC). Religion of households was dichotomized as Muslim vs non-Muslims based on the small number of non-Muslims who were also not Hindu. The Standard of Living Index (SLI) was used as a proxy indicator for characterizing household wealth [26]; a dichotomized wealth variable was created based on SLI scores of 0–50 vs. 51–100 (range 0–100). Women were considered literate based on reporting being able to both read and write in at least one language.

Contact with ASHAs was measured via a single dichotomous variable that assessed whether a woman had any contact with an ASHA during her recent pregnancy. The three outcomes related to health care utilization included receipt of four or more antenatal care (ANC) visits (based on the current WHO standard for adequate ANC) [27], institutional (public or private health facility) vs. home delivery, and current use of any modern contraceptive method. Women who had at least four ANC visits, either at home or at a health facility, during their recent pregnancy were labeled as having had four or more ANC visits. Attendance at any government health facility, privately owned hospital/clinic or an NGO hospital/clinic for the index childbirth was considered to be a facility delivery. Women reporting female/male sterilization or use of an intrauterine device (IUD), oral contraceptive pills, male/female condoms, injectable contraception, implants, diaphragm, foam/jelly or emergency contraceptive pills at the time of survey for purposes of preventing pregnancy were classified as using a modern contraceptive method.

Sample weights calculated based on the multistage sampling design were utilized in all analyses. Chi-square tests were used to evaluate the associations of key predictor variables (caste, religion, wealth categories, literacy) with contact with an ASHA during the recent pregnancy and with the three health service utilization outcomes. Unadjusted logistic regression models further described the relations (Odds Ratio and 95% CI) between these predictors and the health service utilization. These initial analyses assessed a) whether there are socio-economic inequities related to likelihood of being visited by an ASHA, and b) whether such inequities existed among this sample regarding utilization of ANC, facility delivery and family planning, and c) whether being visited by an ASHA related to likelihood of utilizing such care. In order to determine if socio-economic indicators interact with ASHA visits to predict disparities in utilization of care (i.e., whether the effects of ASHA visits on utilization of care differ based on socio-economic status), all socio-economic predictors were entered simultaneously along with ASHA visit in a multivariate logistic regression model for each form of care utilization. Parity and women’s current age were also entered in the model based on their known associations with maternal care utilization. Finally, a multiplicative term statistical interaction between each of the four socio-demographic indicators and ASHA visit was entered into these same models to identify their effect on each health service utilization indicator. The interaction terms were tested using Wald test for interaction and were deemed significant at the level of *p* < 0.05. The nature of significant interactions was further unpacked using stratified logistic regression models. Data were analyzed using STATA 12.0 software (StataCorp, USA).

## Results

As given in Table 1, of the 4912 women who gave birth in last 12 months, 6.8% participated in at least 4 ANC visits during their pregnancy, 62.9% had institutional delivery and 12.6% were using any modern method of family planning during the time of survey. Women belonging to General Caste were more likely to participate in at least four ANC compared to women belonging to other caste categories (14.4% vs. 5.8% for OBC women and 5.5% for SC/ST women, *p* < 0.001). Similar trends were found for institutional delivery and use of modern family planning methods. The proportion of women participating in at least 4 ANC visits, delivering at health facility, and using a modern method of family planning did not differ based on religion. Participation in at least 4 ANC, delivery at a health facility and use of any modern method of family planning was significantly higher among higher vs. lower wealth women. Literate women were more likely to participate in at least 4 ANC visits and to deliver at a health facility as compared to illiterate women, but were not more likely to report use of any modern method of family planning. Women who were giving birth for the first time, were more likely to participate in at least four ANC and deliver at a health facility. However, these women were less likely to use a modern method of family planning as compared to women who had given birth to more than one child. Women, who were below 20 years of age, were more likely to deliver at a health facility and use a modern method of family planning. However, there was no significant difference in the participation of at least 4 ANC based on the age of the mother.

**Table 1:** Sample characteristics and associations with service utilization (*N* = 4912)

	Total Sample (*N* = 4912)	Total Sample (Wtd *N* = 5162)	Any ASHA visit during pregnancy (Wtd % = 46.1; unwtd *n* = 2308)	Service Utilization		
				At least 4 ANC (Wtd % = 6.8; unwtd *n* = 387)	Facility Delivery (Wtd % = 62.9; unwtd *n* = 3215)	Use of any modern method of FP (Wtd % = 12.6; unwtd *n* = 600)
	Unwtd % (n)	Wtd % ^†^ (n)	Wtd % ^†^ [95%CI]	Wtd % ^†^ [95%CI]	Wtd % ^†^ [95%CI]	Wtd % ^†^ [95%CI]
Characteristic						
Caste						
SC/ST	29.2 (1433)	29.7	49.3 [44.3,54.4]	5.5*** [3.6,8.4]	60.3*** [53.4,66.8]	9.6*** [7.7,11.9]
OBC	57.9 (2842)	57.4	44.5 [40.9,48.2]	5.8 [4.6,7.2]	61.8 [56.7,66.7]	12.2 [10.5,14.1]
General	13.0 (637)	12.9	45.9 [40.0,52.0]	14.4 [11.3,18.3]	74.0 [67.2,79.9]	21.0 [16.1,27.0]
Religion						
Non-Muslim	83.4 (4098)	82.0	46.3 [43.2,49.3]	7.0 [5.9,8.3]	63.1 [58.3,67.7]	12.8 [11.2,14.6]
Muslim	16.6 (814)	18.0	45.5 [38.9,52.2]	5.8 [4.4,7.8]	62.2 [51.3,72.0]	11.7 [8.7,15.6]
Wealth index						
Low	80.3 (3946)	82.3	45.8 [42.3,49.3]	5.3*** [4.3,6.5]	59.8*** [54.3,65.1]	10.7*** [9.3,12.1]
High	19.7 (966)	17.7	47.8 [42.9,52.8]	14.0 [11.2,17.5]	77.5 [72.0,82.3]	21.5 [17.0,26.8]
Literacy of woman						
Literate	40.2 (1973)	37.9	47.5 [43.9,51.2]	10.2*** [8.6,12.0]	72.9*** [68.9,76.5]	14.4 [11.8,17.4]
Illiterate	59.8 (2939)	62.1	45.3 [40.7,49.9]	4.8 [3.7,6.1]	56.9 [50.3,63.2]	11.5 [9.7,13.6]
Birth parity						
Primip	29.1 (1431)	28.1	45.4 [40.8,50.2]	9.3* [7.2,11.9]	75.2*** [69.3,80.2]	9.1* [7.3,11.3]
Multip	70.9 (3481)	71.9	46.4 [43.3,49.7]	5.8 [4.7,7.2]	58.2 [52.8,63.4]	13.9 [12.1,16.0]
Age						
Below 20 years	1.6 (76)	1.7	52.5 [39.3,65.4]	6.8 [2.7,16.0]	61.6*** [42.5,77.6]	18.3* [9.4,32.8]
20–24 years	30.8 (1514)	29.1	47.2 [43.2,51.1]	8.1 [6.5,10.1]	69.5 [64.7,73.8]	9.8 [7.9,12.2]
25–29 years	39.4 (1937)	39.9	46.3 [42.3,50.4]	6.6 [5.2,8.3]	63.9 [58.4,69.0]	12.1 [10.0,14.5]
30–34 years	18.2 (895)	19.0	44.7 [37.7,51.9]	5.4 [3.8,7.6]	55.3 [46.7,63.6]	15.7 [12.6,19.4]
35 years and above	10.0 (490)	10.2	44.0 [36.9,51.5]	6.7 [4.0,11.1]	55.2 [47.9,62.2]	15.5 [10.7,21.9]
Any ASHA visits during pregnancy						
Yes	47.0 (2308)	46.1		8.3* [6.8,10.1]	70.7*** [66.4,74.8]	13.8 [11.5,16.4]
No	53.0 (2604)	53.9		5.6 [4.7,6.7]	56.3 [50.2,62.2]	11.6 [10.0,13.3]

^†^ Results presented are weighted to account for sampling design

* Statistically significant at *p* < 0.05

*** Statistically significant at *p* < 0.001

Out of all the women who had delivered in the last 1 year, 46.1% received any ASHA visit during the pregnancy. There was no significant difference in the receipt of any ASHA visit during pregnancy based on demographic characteristics of the households, such as caste, religion, wealth, literacy etc.. Women who reported any contact with an ASHA during their recent pregnancy were more likely to participate in at least 4 ANC visits (8.3%) and institutional delivery (70.7%) as compared to those who did not have any contact with ASHA (5.6%; *p* < 0.01 and 56.3%; *p* < 0.001, respectively). ASHA contact did not relate to likelihood of current use of modern family planning methods. ASHA contact continued to predict increased likelihood of 4 or more ANC visits and facility delivery in adjusted analyses; ASHA visits were not associated with current use of modem family planning methods in multivariate analyses.

In the unadjusted and adjusted regression analysis as given in Table 2, women belonging to SC/ST and OBC castes were less likely, as compared to General Caste women, to participate in at least 4 ANC visits (SC/ST, AOR = 0.49, 95% CI = 0.28, 0.84; OBC, AOR = 0.46, 95% CI = 0.32, 0.65), to deliver at facility (SC/ST, AOR = 0.64, 95% CI = 0.46, 0.88; OBC, AOR = 0.65, 95% CI = 0.50, 0.85) or to be using a modern family planning method at the time of survey (SC/ST, AOR = 0.51, 95% CI = 0.33, 0.78; OBC, AOR = 0.63, 95% CI = 0.46, 0.86). No significant effect of religion was found on any outcome variable. Illiterate women were less likely to receive at least 4 ANC (AOR = 0.60, 95%CI = 0.44, 0.80) and to deliver at a health facility (AOR = 0.63, 95% CI = 0.50, 0.80) relative to literate women. Lower wealth women were less likely to participate in at least 4 ANC (AOR = 0.48, 95% CI = 0.34–0.68), to deliver at facility (AOR = 0.58, 95% CI = 0.42–0.79) and to be using a modern family planning method (AOR = 0.47, 95% CI = 0.34–0.64).

**Table 2:** Unadjusted and adjusted associations of socio-demographics with service utilization (*N* = 4912)

	At least 4 ANC during pregnancy	Facility delivery	Use any modern method of family planning
	(Wtd %^†^ = 6.8)	(Wtd %^†^ = 62.9)	(Wtd %^†^ = 12.6)
Characteristic	OR (95% CI)	OR (95% CI)	OR (95% CI)
Caste			
SC/ST	0.35***(0.20–0.59)	0.53***(0.39–0.72)	0.40***(0.26–0.62)
OBC	0.36***(0.25–0.52)	0.57***(0.44–0.73)	0.52***(0.38–0.70)
General	Ref	Ref	Ref
Religion			
Non-Muslim	Ref	Ref	Ref
Muslim	0.82(0.59–1.15)	0.96(0.65–1.43)	0.91(0.64–1.29)
Wealth index			
Low	0.34***(0.24–0.48)	0.43***(0.32–0.58)	0.44***(0.32–0.60)
High	Ref	Ref	Ref
Literacy of woman			
Illiterate	0.44***(0.33–0.59)	0.49***(0.39–0.62)	0.77(0.57–1.05)
Literate	Ref	Ref	Ref
Any ASHA visits			
No	0.65**(0.50–0.84)	0.53***(0.44–0.64)	0.82(0.64–1.04)
Yes	Ref	Ref	Ref
Caste	AOR^#^ (95% CI)	AOR^#^ (95% CI)	AOR^#^ (95% CI)
SC/ST	0.49*(0.28–0.84)	0.64**(0.46–0.88)	0.51**(0.33–0.78)
OBC	0.46***(0.32–0.65)	0.65**(0.50–0.85)	0.63**(0.46–0.86)
General	Ref	Ref	Ref
Religion			
Non- Muslim	Ref	Ref	Ref
Muslim	0.87(0.54–1.40)	1.05(0.73–1.51)	0.82(0.55–1.20)
Wealth index			
Low	0.48***(0.34–0.68)	0.58**(0.42–0.79)	0.47***(0.34–0.64)
High	Ref	Ref	Ref
Literacy of woman			
Illiterate	0.60**(0.44–0.80)	0.63***(0.50–0.80)	0.82(0.60–1.13)
Literate	Ref	Ref	Ref
Any ASHA visits			
No	0.65**(0.51–0.84)	0.52***(0.43–0.63)	0.82(0.65–1.04)
Yes	Ref	Ref	Ref

† Results presented are weighted to account for sampling design

* Statistically significant at *p* < 0.05

** Statistically significant at *p* < 0.01

*** Statistically significant at *p* < 0.001

^#^ Model adjusted for caste, religion, wealth index, literacy status, number of previous births, age of woman and any ASHA visit during pregnancy

Results of tests for interactions of socio-demographics and contact with ASHA differed across specific socio-demographic variables and health utilization outcomes. No significant interactions between ASHA visits and caste were observed for any of the assessed outcomes. The association of ASHA contact with participation in at least 4 ANC was affected by religion (*p* = 0.02 for interaction term of ASHA visit X religion) as given in Table 3. In stratified logistic regression models for participation in at least 4 ANC, among the Non-Muslim women, any contact with ASHA increases the odds of participating in at least 4 ANC visits (AOR = 1.85, 95% CI = 1.33, 2.58); among Muslim women, no effect of ASHA contact on likelihood of receipt of 4 or more ANC visits was observed.

**Table 3:** Odds ratios for interaction term (socio-demographics and home visit) for service utilization models

	At least 4 ANC during pregnancy
	AOR^#^ (95% CI)
Any ASHA Visit	
Religion	
Non-Muslim	Ref
Muslim	0.44*(0.24–0.83)
No ASHA Visit	
Religion	
Non-Muslim	Ref
Muslim	1.40(0.73–2.68)
Non-Muslim	
Any ASHA visit	1.85***(1.33–2.58)
No ASHA visit	Ref
Muslim	
Any ASHA visit	0.59(0.27–1.30)
No ASHA visit	Ref
Interaction term *p*-value	0.0195

^#^ Model adjusted for caste, religion, wealth index, literacy status, number of previous births, age of woman and any ASHA visit during pregnancy

* Statistically significant at *p* < 0.05

*** Statistically significant at *p* < 0.001

As given in Table 4, the interaction between contact with ASHA during pregnancy and wealth was found to be significant in the models for both participation in at least 4 ANC visits (*p* < .0001) and for delivery at a health facility (*p* < .01). In stratified analyses for participation in ANC, contact with ASHA increased the odds of participation in at least 4 ANC among lower wealth women (AOR = 2.24, 95% CI = 1.66, 3.03). In stratified analyses for facility delivery, contact with ASHA increased the odds or delivering at health facility among women of low wealth (AOR = 2.11, 95% CI = 1.71, 2.60) but not among those in the high wealth category.

**Table 4:** Odds ratios for interaction term (socio-demographics and home visit) for service utilization models

	At least 4 ANC during pregnancy	Facility Delivery
	AOR^#^ (95% CI)	AOR^#^ (95% CI)
Any ASHA Visit		
Wealth Index		
Low	0.79(0.53–1.19)	0.80(0.52–1.23)
High	Ref	Ref
No ASHA Visit		
Wealth Index		
Low	0.27***(0.18–0.42)	0.44***(0.31–0.61)
High	Ref	Ref
Low wealth index		
Any ASHA visit	2.24***(1.66–3.03)	2.11***(1.71–2.60)
No ASHA visit	Ref	Ref
High wealth index		
Any ASHA visit	0.77(0.50–1.18)	1.14(0.77–1.71)
No ASHA visit	Ref	Ref
Interaction term *p*-value	0.0001	0.0095

^#^ Model adjusted for caste, religion, wealth index, literacy status, number of previous births, age of woman and any ASHA visit during pregnancy

*** Statistically significant at *p* < 0.001

Literacy status of woman also modified the association between ASHA visits and likelihood of facility delivery (*p* = .003) (Table 5). In the model limited only to illiterate women, contact with ASHA increased the odds of delivering at a health facility (AOR = 2.34, 95% CI = 1.83, 3.02). Although the odds of facility delivery were also increased based on ASHA contact for literate women (AOR = 1.33, 95% CI = 1.02, 1.71), this effect was significantly smaller than that for illiterate women.

**Table 5:** Odds ratios for interaction term (socio-demographics and home visit) for service utilization Models

	Facility Delivery
	AOR^#^ (95% CI)
Any ASHA Visit	
Literacy	
Literate	1.15(0.85–1.55)
Illiterate	Ref
No ASHA Visit	
Literacy	
Literate	2.03***(1.50–2.74)
Illiterate	Ref
Literate	
Any ASHA visit	1.33*(1.02–1.72)
No ASHA visit	Ref
Illiterate	
Any ASHA visit	2.34***(1.83–3.02)
No ASHA visit	Ref
Interaction term *p*-value	0.0026

^#^ Model adjusted for caste, religion, wealth index, literacy status, number of previous births, age of woman and any ASHA visit during pregnancy

* Statistically significant at *p* < 0.05

*** Statistically significant at *p* < 0.001

## Discussion

Findings of the current study suggest answers to several critical questions regarding the relationship between community health worker visits and socio-demographic health disparities in maternal care and family planning utilization. Consistent with previous studies [28–30], we identified socio-demographic disparities in maternal and reproductive health service utilization. Results of the current study affirm the positive relationship between visits by a community health worker and likelihood of utilizing critical maternal health services; women’s contact with an ASHA during their pregnancy increases their participation in ANC [31] and institutional delivery [32]. However, we did not find any relationship between ASHA contact and use of a modern method of family planning. This is likely due to ASHAs being traditionally focused solely on pregnancy-related care during their visits. The current results underscore the need for provision of additional training and, possibly, a system of incentives to change ASHA behavior to include the required education and advocacy to increase use of modern contraception in UP and nationally.

The current work also sought to answer questions regarding whether ASHAs discriminate in their decisions to visit households based on socio-demographics. Importantly, our observations indicate that the likelihood of visiting a home does not relate to the caste, religion, wealth or literacy of a woman living in that household. Given that ASHA visits relate to increased likelihood of maternal health services utilization (although, not family planning), that more marginalized groups (poorer, illiterate, lower caste) are less likely to utilize these same services, but that the likelihood of an ASHA visit does not vary based on household demographics, it is logical to conclude that either the quality of ASHA visits differ across socio-demographic groups or that the effect of ASHA visits differs across such groups.

To explore the validity of these hypotheses, interaction analyses were conducted to assess whether receipt of ASHA visits interacts with socio-demographics to produce differences in maternal health care utilization. Analysis stratified by woman’s religion demonstrated that contact with an ASHA significantly increases the likelihood of participation in at least four ANC visits among non-Muslim women, but not among Muslim women. This implies either that ASHA visits to Muslim households are of relatively lower quality than those delivered to non-Muslim households, or that the response to, or effect of, ASHA visits differ based on religion. In either case, implications of these findings are that visits to Muslim households should be enhanced in ways that make such households as likely as non-Muslim households to utilize adequate ANC. Training for ASHAs to provide such an enhanced program should address potential biases of health care workers based on religion and how this may influence the quality, quantity and efficacy of their efforts.

Wealth of woman and ASHA contact was found to significantly interact to predict utilization of ANC and delivery at a health facility. A similar interaction effect was observed for delivery at a health facility based on literacy. Across these three interactions, in the absence of an ASHA visit, more marginalized (lower wealth, illiterate) women were less likely to utilize maternal health services. However, in the context of an ASHA visit, the likelihood of utilizing these services rose among the more marginalized groups to a greater extent than it did for the less marginalized (higher wealth, literate), rendering the difference in likelihood of utilization of care insignificant. These findings affirm the utility of ASHA visits in reducing disparities in health care utilization and, presumably, contributing to reductions in health inequities. The findings also highlight that only half of the women in the study geographies had received ASHA visits during their recent pregnancy; intervention to increase the capacity and reach of the ASHA program is required in order to further promote the utilization of critical maternal and reproductive health services and, based on current findings, reduce disparities in this utilization.

The current findings indicate that the effects of ASHA visits, rather than the likelihood of being visited by an ASHA, differ across socio-demographic groups. However, the exact reasons for these differences in the impact of ASHA visits remain to be clarified. One possibility is that the quality of home visits by ASHAs (e.g. time spent, content of interaction, use of job-aids for counselling etc.) differs based on the different demographic profile of households. The number of ASHA visits may also differ across groups, which is an important question for future research. Another possibility for differential impact of home visits by ASHAs leading to inequities in health service utilization is the variation in how the information imparted by an ASHA may or may not factor into the health services seeking decision-making and behavior of households. Currently, a key policy mandate in India is to include the right to health as a fundamental right. This would define inadequate quality of health services as an important violation of rights, along with unavailability or denial of health services. To ensure universal health coverage, CHWs play a major role. However, given the disparities in service delivery outcomes based on apparent differences in effects of CHW visits based on household demographic and economic characteristics, additional policies to improve utilization of critical maternal and reproductive health services among the most marginalized in Indian society are needed.

Although the current findings provide important insights into the role of community health workers in India in addressing health disparities, there are several limitations related to the current design that are worthy of note. One limitation is that the data were collected solely from ‘high priority districts’, which are not representative of the state as a whole. It is also possible that biases related to self-reported data, including recall bias, may have affected the reliability of reports of health care receipt and utilization. The potential for such bias is likely reduced based on the recall period from delivery to data collection being 11 months or less. Finally, the current analyses involve cross-sectional data, thus causality cannot be inferred.

## Conclusion

In conclusion, the ASHA visits play a vital role in promoting utilization of critical maternal health services, more so among the most disadvantaged sub-groups. And that inequities exist in service utilization both in the presence/absence of ASHA visits. To determine the mechanisms underlying socio-demographic inequities in effects of household visits by ASHA, further research, both quantitative and qualitative, is required. It is also recommended that demographics and other equity indicators are included in standard data collected in health information systems to allow monitoring of inequities and inform related intervention efforts in geographies with high maternal and child mortality.

## Abbreviations

ANC: Antenatal care
AOR: Adjusted odds ratio
ASHA: Accredited social health activist
CHW: Community health workers
CI: Confidence interval
CPR: Contraceptive prevalence rate
GC: General caste
HPD: High priority district
IMR: Infant mortality rate
IUD: Intrauterine device
NGO: Non-Governmental Organization
NMR: Neonatal mortality rate
OBC: Other backward caste
SC/ST: Schedule caste/schedule tribe
SLI: Standard of living
TFR: Total fertility rate
UP: Uttar Pradesh
UPTSU: Uttar Pradesh Technical Support Unit
Wtd.: Weighted
